# Evaluation of Antibacterial Efficacy of Centella asiatica-Mediated Selenium Oxide Nanoparticles Against Multidrug-Resistant Upper Respiratory Isolates

**DOI:** 10.7759/cureus.58350

**Published:** 2024-04-15

**Authors:** Mounithaa Nataraj, Durai Singh Carmelin, P Geetha Sravanthy, Muthupandian Saravanan

**Affiliations:** 1 Department of Pharmacology, Saveetha Dental College and Hospitals, Saveetha Institute of Medical and Technical Sciences, Chennai, IND

**Keywords:** multidrug resistance, nanobiotechnology, antibacterial activity, selenium oxide nanoparticles, green synthesis

## Abstract

Background

The evolution of new respiratory diseases, especially upper respiratory tract infections and resistance of pathogens to various antibiotic treatments, needs an alternative way of medication. Chronic respiratory infections in both adults and infants are the major cause of morbidity and mortality, particularly in developing countries. The widespread application of nanomaterials in the field of medicine and the incorporation of nanoparticles in drugs are taken into account. These nanomaterials are involved along with the biosynthesis of plant extract. In this study, selenium oxide nanoparticles (SeO-NPs), known as a significant trace element for human health, were synthesized in an eco-friendly manner.

Methodology

Green synthesis of *Centella asiatica*-mediated SeO-NPs was proceeded by titration method and nanoparticles were synthesized. The color intensity, morphological characters, functional properties, and involvement of phytochemical compounds were studied by using UV-visible spectroscopy (UV-Vis), Fourier-transform infrared spectroscopy (FT-IR), X-ray powder diffraction (XRD), scanning electron microscope (SEM), and energy-dispersive X-ray spectroscopy (EDX) analysis.

Results

The synthesized extract showed a color change from brown to ruby red. Results obtained by characterization and biological assays depicted that the Centella asiatica-mediated SeO-NPs showed absorbance at the peak level 320 nm by UV-Vis spectroscopy, several phytochemical compounds, and O-H functional groups by FT-IR which may be involved in the reduction of the selenium oxide nanoparticles. The XRD showed 57.1% crystalline and 42.6% amorphous nature. The SEM images showed that agglomerated spherical shapes were involved in biological activities. The EDX analysis showed the presence of Se, C, and O compounds. Further, the antibacterial activity of the synthesized nanoparticles showed significant activity in the multidrug-resistant respiratory pathogens.

Conclusions

Based on the characterization studies and biomedical assays, it can be concluded that the incorporation of SeO-NPs along with the plant extract serves as the best remedy and organic treatment for upper respiratory tract infections. We plan to conduct further in-vivo, toxicity-level studies, and clinical trials.

## Introduction

The emergence of new diseases and the virulence of the pathogens create fear because of the challenges in the treatment of diseases to find an effective cure [1]. Antibiotics have been discovered and utilized as treatments for various diseases caused by microbial pathogens. However, the effectiveness of certain antibiotics in treating these respiratory diseases has diminished or ceased altogether due to the development of pathogen resistance [2]. Multidrug-resistant bacteria are microorganisms that have acquired resistance to multiple antimicrobial agents from various categories [3]. In the United States, the Centers for Disease Control and Prevention (CDC) has estimated that multidrug-resistant pathogens are responsible for nearly 2 million illnesses and approximately 23,000 deaths per year [4]. The prevalence of multidrug-resistant pathogens commonly begins with oral and respiratory infections. The potential reservoir for respiratory pathogens is the oral cavity, which is directly connected anatomically to the respiratory tract [5]. Respiratory infection is the primary cause of death in children under the age of five, accounting for more than 20% of the annual global mortality rate of 10.6 million [6]. Among the respiratory tract infections, more attention was given to the upper respiratory tract infection. The upper respiratory tract harbors a well-documented bacterial community, known as the microbiome, which resides in the nasal cavity and nasopharynx. Within this microbiome, there are opportunistic bacterial pathogens that are transient and can cause illness when they invade other host tissues. Respiratory tract infections caused by these opportunistic pathogens contribute significantly to the overall burden of disease [7]. To overcome infection and challenges in treating multidrug-resistant bacterial communities, alternative methods of treatment should be incorporated in an eco-friendly manner.

Plants are commonly utilized in the development of new medicinal formulations due to their abundant secondary metabolites. These secondary metabolites, also known as phytochemicals, act as a natural defense mechanism against diseases and pathogens. The use of these phytochemical antibiotics is advantageous due to their minimal to no side effects in comparison to synthetic antibiotics. Additionally, they possess the ability to overcome pathogen resistance to existing antibiotics [8]. The exploration of medicinal plants has proven valuable in the quest for discovering novel biologically active compounds through research [9]. *Centella asiatica* L is a perennial herbaceous creeper belonging to the Umbeliferae family. It thrives in moist environments and is found extensively in tropical and subtropical regions across various countries. Secondary metabolites such as triterpenoids, volatile and fatty acids, alkaloids, glycosides, flavonoids, vitamins B, C, G, and some other amino acids are found in this plant [10].

Treatment has to be provided which cures rapidly and effectively with the help of the novel therapeutic methods. Nanotechnology has emerged as a highly promising method for creating nanomaterials with distinct properties at the nanometer scale [11]. It holds great potential in various domains, such as medicine, biotechnology, chemistry, and physics, offering new possibilities and capabilities [12,13]. Among the various nanoparticles, selenium oxide nanoparticles (SeO-NPs) are found in human and animal bodies in low concentrations and are known for their significant activity [14]. The properties of SeO-NPs vary depending on factors such as concentration, temperature, biomolecule nature, and pH of the reaction mixture. As a result, SeO-NPs can exist in various crystalline and amorphous forms, each with its own shape, size, and structure. It is worth noting that the size and shape of SeO-NPs play a significant role in determining their properties. For instance, research has shown that SeO-NPs exhibit high biological activity and low toxicity [15,16]. Recently, there has been a growing focus on research dedicated to the synthesis of nanoscale metals using a variety of methods, including chemical, physical, and environmentally friendly synthesis approaches [17]. Of these approaches, phyto-nanotechnology has emerged as a promising approach for synthesizing nanoparticles, offering environmentally friendly, straightforward, fast, stable, and cost-effective methods [18]. Typically, the extraction process involves immersing ground plants in a solvent, while maintaining appropriate environmental conditions. It is important to note that different green substances have varying optimal conditions for extraction [19]. This study focuses mainly on upper respiratory tract infections to overcome the multidrug resistance conditions in an environmentally safe and beneficial manner aided by the *Centella asiatica-*mediated synthesized SeO-NPs depending upon their biological activities.

## Materials and methods

Materials and bacterial strains

Selenium oxide, Muller-Hinton agar (MHA), streptomycin, and dimethyl sulfoxide (DMSO) were purchased from Hi Media India. Upper respiratory isolates of Methicillin-resistant *Staphylococcus aureus* (MRSA), *Enterococcus faecalis*,* Pseudomonas aeruginosa*, and *Klebsiella pneumoniae* were obtained from the Medical Microbiology Laboratory at Saveetha Medical College in Chennai.

Sampling

*Centella asiatica* leaves were collected from Poonamallee, Chennai, Tamil Nadu, and Dr. N. Siva, Assistant Professor, Department of Botany, Raja Doraisingam Government Arts College Sivagangai, Tamil Nadu, authenticated the sample’s taxonomic identification.

Green synthesis of *Centella asiatica* leaf extract-mediated SeO-NPs

Fresh leaves of *Centella asiatica* were collected and thoroughly cleaned. The well-cleaned leaves were air-dried and powdered using a mechanical grinder (Nanchang Kay Xin Yue Technologies Co., Jiangxi, China). To prepare the aqueous plant extract, 10 g of the powdered leaves was added to 200 mL of distilled water. The prepared solution was autoclaved at 121°C for 20 minutes and then allowed to cool. Then, the aqueous extract was filtered with Whatman filter paper. Synthesis of SeO-NPs mediated by *Centella asiatica* aqueous extract was carried out by titration method. For this, 0.01 M of SeO was taken in a burette, and the conical flask was taken with aqueous *Centella asiatica* leaf extract and placed in a magnetic stirrer. The SeO was added dropwise to the plant extract. This mixture solution was taken in an Eppendorf tube (T0) and exposed to ultraviolet (UV) light for one hour before keeping it in a shaker overnight. The reaction mixture was stirred for a designated period to facilitate the reduction of selenium ions and the formation of SeO-NPs. Following this, sample 2 (T24) was subjected to UV and centrifuged at 4,500 rpm for 30 minutes. The supernatant was discarded, distilled water was added to the remaining pellet and mixed thoroughly, and then recentrifuged at 4,500 rpm for 15 minutes. The supernatant was discarded and the pellet was transferred and desiccated in a petri dish and placed in a hot air oven set at 60°C overnight. The dried material was scraped into a fine powder, facilitating the synthesis of SeO-NPs.

Characterization of SeO-NPs

Characterization was performed to confirm the presence of specific nanoparticles synthesized by various analysis techniques such as UV-visible (UV-Vis) spectroscopy (Labman Double Beam UV-vis spectrophotometer LMSPUV1900S, India, 190-1,100 nm), Fourier-transform infrared spectroscopy (FT-IR) (Bruker Alpha II, Germany), X-ray powder diffraction (XRD) (Bruker D8 Advance, Germany), scanning electron microscope (SEM) (JEOL-800S), and energy-dispersive X-ray spectroscopy (EDX) (OXFORD X-Plor-30/C-Swift). The UV-Vis spectroscopy aided in examining the optical characteristics of SeO-NPs synthesized with *Centella asiatica* leaf extract. Further, the presence of chemical composition was analyzed by FT-IR spectroscopy. The XRD facilitated identifying whether the present SeO-NPs were in a particular crystalline phase or an amorphous state. Characteristic diffraction patterns are displayed by each crystalline and amorphous phase. The SEM-EDX analysis helped confirm the morphological and elemental composition present in the synthesized SeO-NPs.

Antibacterial activity

The antimicrobial activity was assessed using the well diffusion method [20]. MHA plates were prepared and using sterile swabs, the bacterial cultures MRSA, *Enterococcus faecalis*,* Pseudomonas aeruginosa*,and* Klebsiella pneumoniae* were evenly swabbed on the agar plates with the corresponding microbial suspension. Using a sterile tip, wells were aseptically bored into the agar plates. Different concentrations of *Centella asiatica* SeO-NPs extract were dispensed into the wells, with one well serving as the negative control, DMSO (30 µL), and another one as the positive control, streptomycin (30 µL). After about 24 hours, the zone of inhibition was measured using a calibrated ruler (zone scale).

## Results

Green synthesis of SeO-NPs

**Figure 1:**
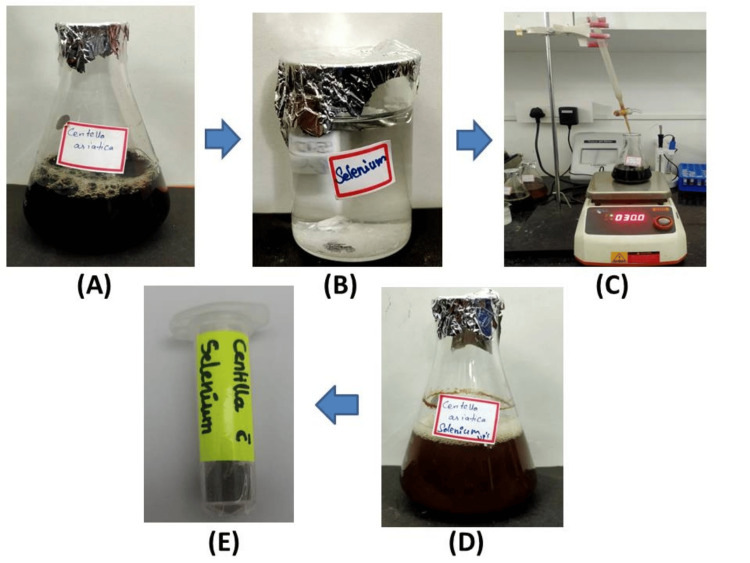
Overview of synthesis of Centella asiatica-mediated SeO-NPs. (A) 150 mL aqueous extract of Centella asiatica. (B) 25 mM of SeO. (C) Titration process. (D) Synthesized SeO-NPs. (E) The powdered form of SeO-NPs. SeO = selenium oxide; SeO-NPs = selenium oxide nanoparticles

*Centella asiatica*-mediated SeO-NP extract was synthesized using the titration method. The synthesized extract showed a color change from black to brown (Figure 1). This color change indicates the reduction of SeO to SeO-NPs due to the presence of secondary metabolites in the synthesized leaf extract.

UV-visible spectroscopy

**Figure 2:**
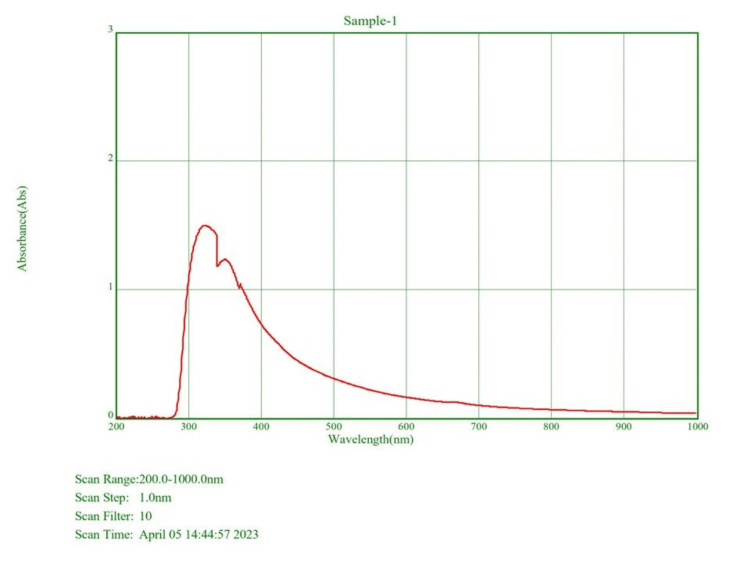
UV-visible spectroscopy absorbance wavelength range of Centella asiatica-mediated SeO-NPs. SeO-NPs = selenium oxide nanoparticles

The SeO-NPs synthesized through *Centella asiatica* exhibited a distinct absorption band at approximately 320 nm, indicating the formation of SeO-NPs through surface plasma resonance, as depicted in Figure 2.

FT-IRspectroscopy

**Figure 3:**
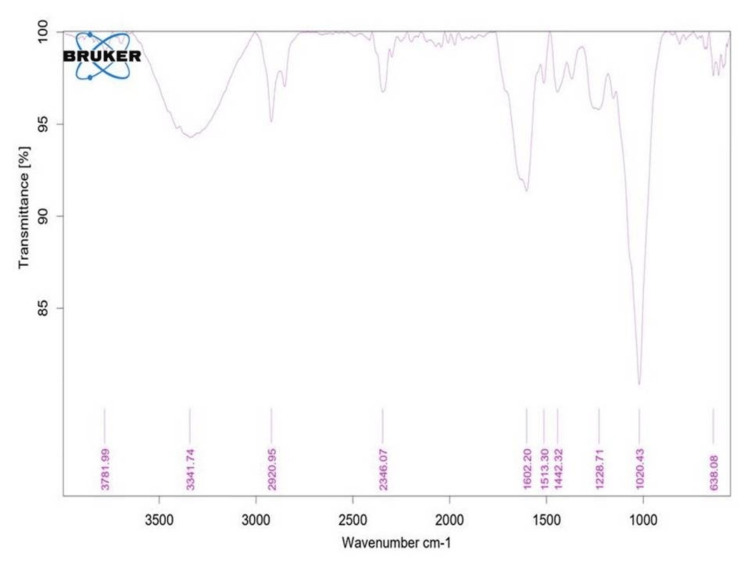
Fourier-transform infrared spectroscopy analysis of chemical compounds and functional groups within the 500-4,000 cm-1.

FT-IR spectroscopy was utilized to characterize the synthesized SeO-NPs and identify the functional groups present in the nanocomposites. Our findings indicated the presence of more than five distinct functional groups in the synthesized nanocomposites. Notably, peaks were observed at 3,341 cm^-1^, 1,602 cm^-1^, 1,513 cm^-1^, 1,442 cm^-1^, 1,228 cm^-1^, 1,020 cm^-1^,^ ^and 638 cm^-1^. These peaks are attributed to various functional groups such as O-H stretching, C=C, alkanes, C-O-C, C-CI, and C-Br, respectively, as illustrated in Figure 3.

XRD analysis

**Figure 4:**
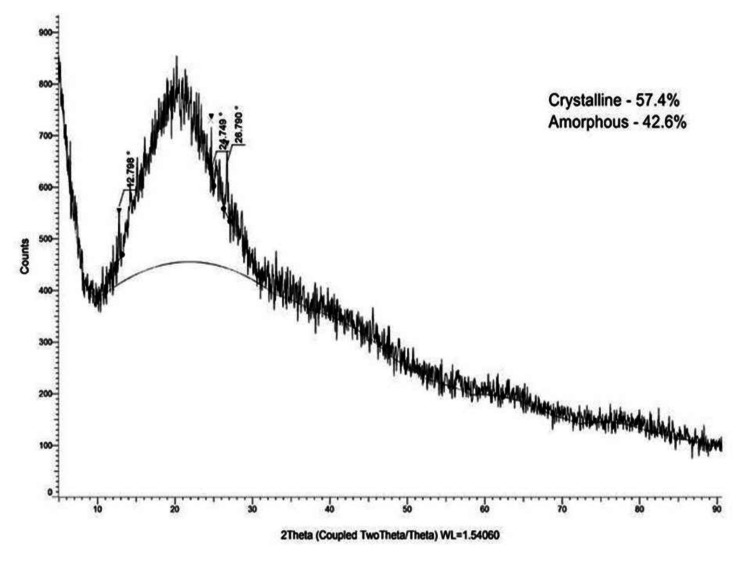
Energy-dispersive X-ray spectroscopy of Centella asiatica-mediated SeO-NPs. SeO-NPs = selenium oxide nanoparticles

The synthesized SeO-NPs exhibited a higher degree of crystallinity (57.4%) compared to amorphous content (42.6%). Consequently, the synthesized SeO-NPs demonstrated a notably stable crystalline nature, as depicted in Figure 4.

SEM

**Figure 5:**
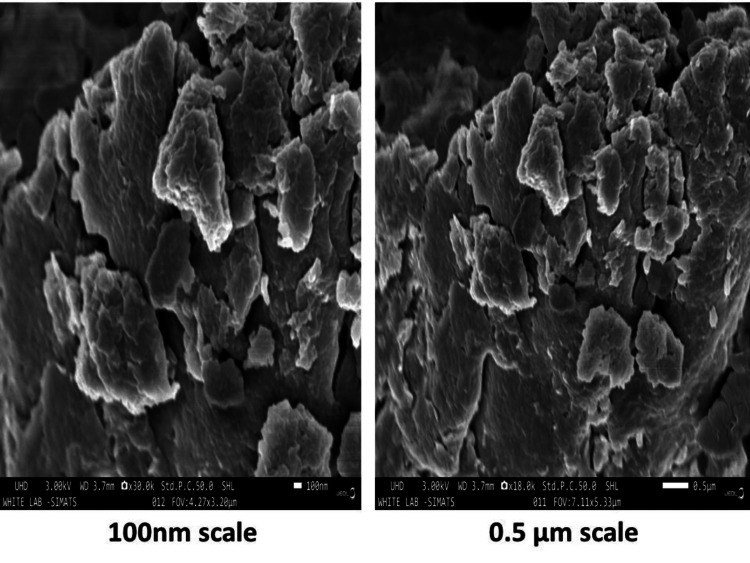
Scanning electron microscope analysis of Centella asiatica-mediated SeO-NPs at 0.5 µm and 100 µm. SeO-NPs = selenium oxide nanoparticles

The SEM analysis showed the morphological characteristics of the synthesized *Centella asiatica*-*mediated* SeO-NPs which were found to be agglomerated spherical shapes distributed with an average particle size of 80-100 nm (Figure 5).

EDX spectroscopy

**Figure 6:**
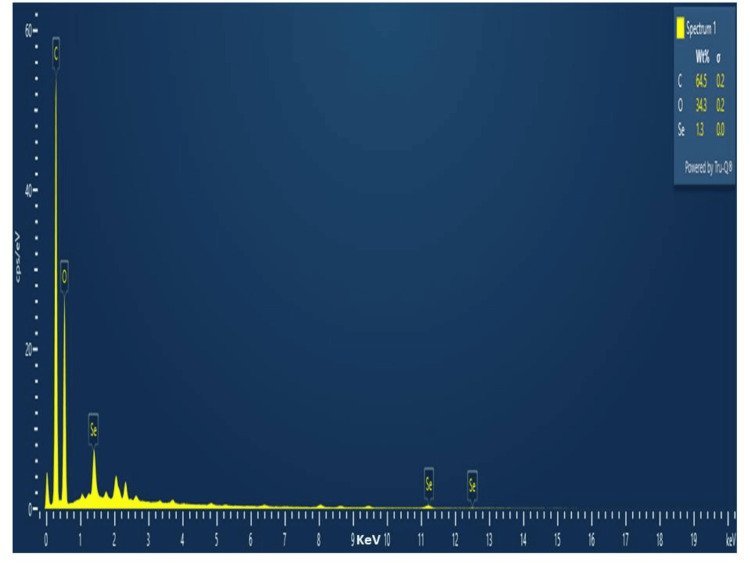
Energy dispersive X-ray spectroscopy analysis of SeO-NPs present in Centella asiatica-mediated SeO-NPs. SeO-NPs = selenium oxide nanoparticles

The EDX analysis revealed the elemental composition of the synthesized nanoparticles in which C, O, and Se were present in 64.5%, 34.3%, and 1.3%, respectively, as shown in Figure 6.

Antibacterial activity

**Figure 7:**
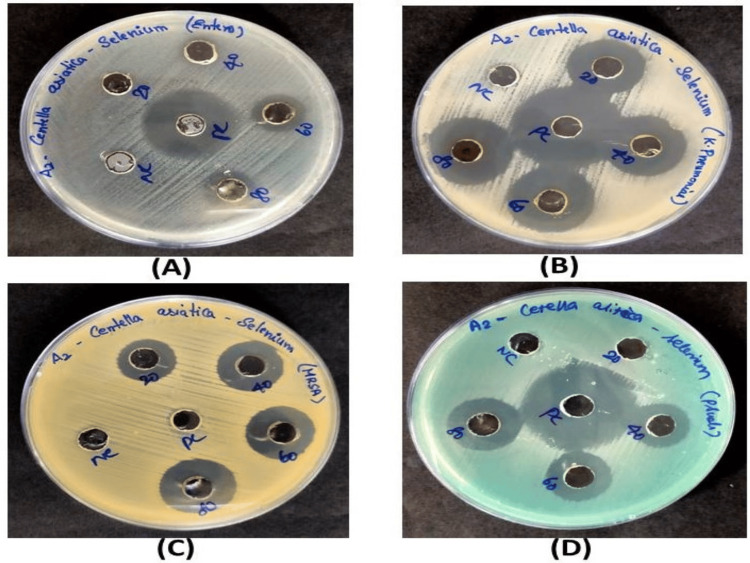
Antibacterial activity of Centella asiatica-mediated SeO-NPs against multidrug-resistant pathogens. (A) *Enterococcus faecalis*, (B) *Klebsiella pneumoniae*, (C) methicillin-resistant *Staphylococcus aureus*, (D) *Pseudomonas aeruginosa* and their zone of inhibition. SeO-NPs = selenium oxide nanoparticles

The *Centella asiatica*-*mediated* SeO-NPs showed significant antibacterial activity against MRSA, *Klebsiella pneumoniae*,* Pseudomonas aeruginosa*, and *Enterococcus faecalis* showing moderate activity at 80 µg/mL concentrations when compared to the standard. The zone of inhibition is shown in Figure 7.

## Discussion

The green synthesis of the *Centella asiatica*-mediated SeO-NPs showed a color change from black to brown, which can be compared with a similar study, wherein the synthesis of Se-NPs mediated by *Ceropegia bulbosa Roxb* extract showed a color change from yellow to ruby red [21]. Hence, various biomolecules found in plants, such as proteins, enzymes, flavonoids, terpenoids, phenolic compounds, and organic acids, among other compounds, are involved in the synthesis and packaging of nanoparticles [22].

The UV-Vis spectroscopy of *Centella asiatica*-mediated SeO-NPs showed a wavelength range of absorption peak at 320 nm. Similarly, the UV-Vis spectroscopy of synthesized Se-NPs from extracts of *Withania somnifera *showed an absorption peak range of 320 nm [23]. Thus, this comparative absorption peak confirms the presence of SeO-NPs. The FT-IR analysis of our leaves extract mediated SeO-NPs showed a broad peak of 3,341.74 cm^-1 ^related to the stretching vibration of O-H groups. In comparison, Se-NPs synthesized based on *Amphipterygium glaucum* extract showed peaks of the functional group, especially a broad absorption peak of 3,256 cm^-1^ responsible for the stretching vibrations (O-H) group [24]. Similar to another previous study, the Se-NPs synthesized from orange peel extract showed a range of absorption at 3,279.53 cm^-1^ with the stretching vibration of the O-H group [25]. Hence, the presence of the O-H group may be responsible for the reduction of SeO-NPs. The analysis of XRD for *Centella asiatica*-mediated SeO-NPs showed diffraction peaks of 2Ɵ 24.749° compared to the synthesis of Se-NP with orange peel extract showed similar diffraction planes 23.8° [25], which denotes the presence of crystalline nature SeO-NPs. In comparison to the Se-NPs synthesized from *Withania somnifera*, the SEM analysis showed an agglomerated spherical shape [23]. Compared to this study, our *Centella asiatica*-mediated synthesized SeO-NPs showed a similar agglomerated spherical shape. This agglomerated spherical shape may be due to the presence and binding of a large number of functional groups. Some studies have shown that these agglomerated spherical shapes exhibited significant biological activity [26,27]. The EDX analysis showed the presence of Se, C, and O which is similar to the study of orange peel extract synthesis of Se-NPs [25].

The antibacterial activity for the respective multidrug-resistant bacteria *Klebsiella pneumonia *was sensitive to the *Centella asiatica*-mediated SeO-NPs and showed an inhibition zone of 22 mm at 20 µg/mL whereas a similar study of green synthesized Se-NPs from *Elaeagnus indica* leaf extract showed a minimum zone of inhibition of 10 mm at 25 µg/mL concentration [28]. In comparison to this study, the *Centella asiatica*-mediated SeO-NPs showed significant antibacterial activity against the upper respiratory tract infective pathogens. We aim to conduct further in-vivo studies and clinical trials in the future depending on their activity.

Limitations

This study presents various in-vitro analyses to assess the synthesized SeO-NPs using *Centella asiatica *leaf extract. Further exploration through in-vivo research, including animal and clinical trials, would enhance our understanding of its effects.

## Conclusions

The study found that Centella asiatica-mediated SeO-NPs had crystalline and amorphous properties, an agglomerated spherical shape, and demonstrated significant antibacterial activity against multidrug-resistant bacteria, particularly upper respiratory tract isolates. This combination of eco-friendly, plant extract-mediated, synthesis of nanoparticles can be made into a pharmaceutical product. Further validation will include minimum inhibitory concentration, minimum bactericidal concentration, in vivo, and toxicological studies.
